# Assessment of the educational value of robotic‑assisted thoracoscopic pulmonary lobectomy videos

**DOI:** 10.20452/wiitm.2025.17994

**Published:** 2025-11-25

**Authors:** Nilay Çavuşoğlu Yalçın, Ayşegül Güler, Muharrem Özkaya

**Affiliations:** Department of Thoracic Surgery University of Health Sciences Antalya Training and Research Hospital Turkey

**Keywords:** Critical View of Safety, Laparoscopic Surgery Video Educational Guidelines, robotic­ ‑assisted thoracic surgery, surgical education, video assessment

## Abstract

**INTRODUCTION:**

Robotic-assisted thoracic surgery (RATS) is increasingly used in lung cancer treatment. As surgical education progressively shifts to online platforms, such as YouTube, concerns have emerged regarding the reliability of available content. This study evaluated the educational quality of the most-viewed RATS lobectomy videos on YouTube, using the Laparoscopic Surgery Video Educational Guidelines (LAP-VEGaS) and the Critical View of Safety (CVS) criteria.

**AIM:**

We aimed to evaluate the educational quality of widely viewed YouTube videos on RATS using the LAP-VEGaS assessment and CVS criteria tools.

**MATERIALS AND METHODS:**

A YouTube search was performed using a key word “robotic lobectomy.” A total of 25 videos with more than 5000 views that met the inclusion criteria were evaluated in terms of video characteristics and educational quality. The assessment was performed using the CVS and LAP-VEGaS criteria, and statistical analyses were conducted to explore correlations between video features and the scores.

**RESULTS:**

A total of 25 videos met the inclusion criteria. Right upper lobectomy was the most frequently demonstrated procedure. Median view count was 7157 (6001–14 152), with significant correlations between views and likes, as well as duration online. Overall educational quality was limited, with median CVS compliance of 50% (50%–62.5%) and a median LAP-VEGaS score of (4–10.5).

**CONCLUSIONS:**

The educational quality of robotic-assisted lobectomy videos on YouTube is heterogeneous and generally suboptimal. Peer-reviewed and standardized video archives curated by academic institutions or professional societies are needed to ensure reliable resources for robotic surgery training.

## INTRODUCTION 

Lung cancer is the most frequently diagnosed malignancy worldwide, and the leading cause of cancer-related mortality.^1^

In early-stage non-small cell lung cancer, lobectomy combined with lymph node dissection remains the gold-standard surgical approach, offering curative potential and achieving 5 -year survival rates of up to 95%.^2^^,^^3^^,^^4^

Over the past 3 decades, advances in surgical techniques and medical technologies have accelerated the adoption of minimally-invasive approaches. In comparison with conventional open surgery, video-assisted thoracoscopic surgery (VATS) has represented a significant advancement in thoracic surgery; however, over time, its inherent limitations-such as 2 -dimensional and restricted visualization, ergonomically suboptimal rigid instruments, and limited maneuverability in confined spaces-have become increasingly apparent.

To overcome these constraints, robotic surgical systems have entered a new stage in the evolution of minimally-invasive surgery. Robotic-assisted thoracic surgery (RATS) provides surgeons with high-definition 3-dimensional visualization, advanced wrist-like instrument articulation, and superior ergonomics, thereby overcoming many of the limitations associated with VATS.^5^^,^^6^

The clinical journey of RATS began in 2002, when reported first robotic lung resections were reported. 7 Since then, various resection approaches using the da Vinci Surgical System (Intuitive Surgical Inc., Sunnyvale, California, United States) have been adopted by thoracic surgeons worldwide.^2^

**FIGURE 1:**
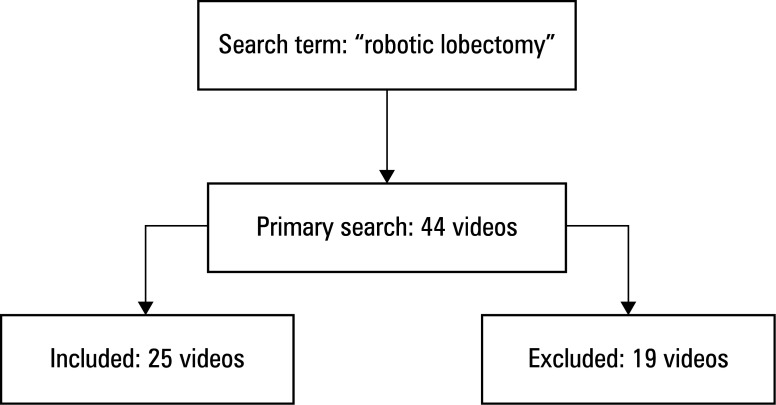
Video selection flow diagram

**FIGURE 2:**
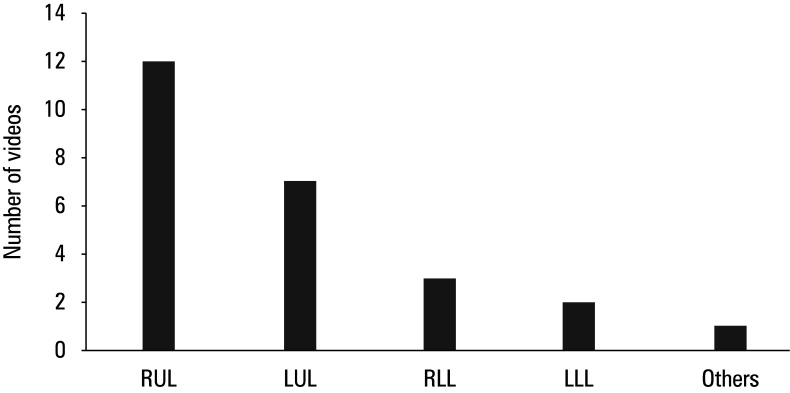
Distribution of the resected lobes in the analyzed videos

Abbreviations: LLL, left lower lobe; LUL, left upper lobe; RLL, right lower lobe; RUL, right upper lobe

Today, robotic surgery-driven by rapid technological advances and their integration into surgical practice-has not only achieved widespread use in the treatment of mediastinal and pulmonary lesions, but has also emerged as a key modality representing the future of surgical approaches.^8^

With the widespread adoption of minimally--invasive thoracic techniques, surgical education has undergone a parallel transformation. Traditionally based on operating room experience and didactic instruction, training has become increasingly supported by digital platforms. Among the most accessible video-sharing websites, YouTube (Google, Mountain View, California, United States) is a popular resource for surgical trainees worldwide.^9^

However, concerns have been raised regarding the heterogeneity of online content, the absence of peer review, and the variability in educational quality. Studies across different surgical disciplines have highlighted that, although YouTube videos can serve as a valuable adjunct learning tool, their quality often fails to meet standardized educational criteria. ^10^

In this context, an international and multidisciplinary panel of instructors and trainees developed the Laparoscopic Surgery Video Educational Guidelines (LAP-VEGaS) consensus statement to evaluate the educational value of laparoscopic surgery videos.^11^

## AIM 

This article aimed to evaluate the educational quality of the most-viewed RATS lobectomy videos on YouTube, using the LAP-VEGaS and Critical View of Safety (CVS) criteria.

## MATERIALS AND METHODS 

A search was performed on the YouTube platform on July 27, 2025, using a key word "robotic lobectomy." No alternative terms, synonyms, or Boolean operators were applied. To minimize algorithm-dependent personalization, the search was performed while signed out of any user account (incognito mode), after clearing browser cache and cookies. Default YouTube settings were preserved, including automatic location detection and language preferences; no manual adjustments were made to regional or language filters. A total of 44 videos with more than 5000 views were initially identified. In our study, the view threshold of 5000 or more views was adopted as a pragmatic visibility criterion to exclude videos with limited exposure or those not yet widely disseminated. Each video was independently reviewed by 2 thoracic surgeons to assess eligibility and relevance. Videos in the form of cartoons / animations, illustrations, or published in languages other than English were not taken into account. Based on these criteria, 19 videos were excluded and 25 videos were included in the final analysis (FIGURE 1).

For each video, the following variables were recorded: title, URL, type of lobectomy, number of views, duration of online availability (days), video length (minutes), image quality (resolution in pixels), number of likes, number of subscribers, uploader type, and country of origin.

Image quality was categorized based on the resolution reported on the YouTube platform as low ( 480 pixels), medium ( 720 pixels), or high (1080 pixels). No subjective visual scoring scale was used.

### Statistical analysis 

All statistical analyses were conducted using SPSS Statistics software, version 27.0 (IBM, Armonk, New York, United States). Two board-certified thoracic surgeons independently (NÇY and MÖ) evaluated all videos after a joint calibration session to ensure consistent interpretation of the scoring criteria. Interobserver agreement of κ = 0.84 was calculated using the Cohen κ coefficient, indicating excellent reliability. Any discrepancies between the raters were resolved by consensus.

The videos were evaluated for compliance with the LAP-VEGaS and CVS frameworks.^11^ As there is no universally accepted definition of the CVS in robotic lobectomy, the stages proposed by Li et al^12^ and the CVS criteria developed by Balta et al^13^ were adopted. For the CVS assessment, 8 criteria were used, with noncompliance scored as 0 and compliance as 1. For the LAP-VEGaS assessment, 9 criteria were applied, where noncompliance was scored as 0 , partial compliance as 1 , and full compliance as 2 .

**TABLE 1:** Main characteristics of the analyzed videos

Rank	URL	Title	Upload date	Access date	Lobe	No. of views	No. of likes	No. of days online	Length, min	Image quality, pix	No. of subscribers	Uploader type	Country of origin	LAP-VEGaS score	LAP-VEGaS, %	CVS score	CVS, %
1	https://www.youtube.com/watch?v=n85q2ix1C_Q	Robotic-Assisted Left Upper Lobectomy in Non-Small Cell Lung Cancer With N1 Disease	April 24, 2018	July 27, 2025	LUL	108180	724	2620	12	1080	78000	Professional society	United States	11	61	4	50
2	https://www.youtube.com/watch?v=28FuV1M3YK4	Robotic Lobectomy Lung Surgery performed by Surgeon Albert DiMeo St. Vincent's.mov	January 30, 2013	July 27, 2025	RUL	94563	437	4560	12	480	2210	Hospi-tal/academic institution	United States	10	56	5	62.5
3	https://www.youtube.com/watch?v=auG1TJoooIY	daVinci Robotic Pulmonary Left Upper Lobectomy for Lung Cancer Treatment VATS	February 9, 2012	July 27, 2025	LUL	75672	128	4917	14	720	302	Individual surgeon	Unknown	3	17	4	50
4	https://www.youtube.com/watch?v=rbGDffcJfT8	Robotic Lobectomy | Brigham and Women's Hospital	December 11, 2018	July 27, 2025	RUL	52501	305	2419	14	480	60100	Hospi-tal/academic institution	United States	10	56	4	50
5	https://www.youtube.com/watch?v=UCOkT_EgJTI	Dr. Robert J. Cerfolio: Left Lung Lower Lobectomy	June 9, 2015	July 27, 2025	LLL	23575	96	3701	23	720	7260	Medical industry	India	5	28	5	62.5
6	https://www.youtube.com/watch?v=YrdjzsfAtNo	Lung Cancer | How to do Robotic Lung Cancer Surgery | Thoracotomy Surgery for Lung Cancer	February 26, 2018	July 27, 2025	RUL	14126	55	2705	8	1080	161000	Medical industry	Unknown	3	17	4	50
7	https://www.youtube.com/watch?v=R5vTz-9Kx	Robotic Left Upper Lobectomy	March 7, 2018	July 27, 2025	LUL	14150	65	2699	10	1080	78000	Professional society	United States	11	61	3	37.5
8	https://www.youtube.com/watch?v=qJSAbDkyYcs	Robotic Lobectomy in 18 Minutes: Step-by-Step, LLLobectomy	November 29, 2018	July 27, 2025	LLL	14152	0	2431	18	720	1790	Individual surgeon	Unknown	9	50	5	62.5
9	https://www.youtube.com/watch?v=xH-XE75fu	Robotic Xi Right Upper Lobectomy	March 14, 2016	July 27, 2025	RUL	12825	39	3423	33	1080	1240	Individual surgeon	Unknown	3	17	4	50
10	https://www.youtube.com/watch?v=CnW7-mDJH	Robotic Right Upper Lobectomy	July 2, 2018	July 27, 2025	RUL	10414	40	2581	15	1080	78000	Professional society	England	7	39	5	62.5
11	https://www.youtube.com/watch?v=z_YiKNu2n-k	Deconstructed Xi Robotic Right Upper Lobectomy in 10 Steps	July 2, 2018	July 27, 2025	RUL	9989	101	2581	33	1080	519	Individual surgeon	United States	10	56	4	50
12	https://www.youtube.com/watch?v=4ge9cmDcguo	Robotic Xi Right Lower Lobectomy	January 27, 2017	July 27, 2025	RLL	7794	25	3103	41	720	1240	Individual surgeon	Unknown	3	17	4	50
13	https://www.youtube.com/watch?v=dRONEFz4xE0	Robotic Lobectomy using a Sub-Xiphoid Approach	May 10, 2016	July 27, 2025	RUL	7157	27	3366	2	480	78000	Professional society	Unknown	7	39	2	25
14	https://www.youtube.com/watch?v=87krAsRaIRs	Robotic-assisted thoracoscopic surgery (RATS) lobectomy	October 13, 2020	July 27, 2025	RUL	7150	47	1748	12	720	21800	Professional society	United States	14	78	5	62.5
15	https://www.youtube.com/watch?v=YujFZbsAo_Q	Robotic Lobectomy in 18 Minutes: Step-by-Step, RLLobectomy	October 23, 2018	July 27, 2025	RLL	6704	0	2469	18	720	1790	Individual surgeon	Unknown	7	39	5	62.5
16	https://www.youtube.com/watch?v=9SpG08BHpvE	Robotic Right Upper Lobectomy	April 13, 2018	July 27, 2025	RUL	6499	30	2662	23	720	1240	Individual surgeon	Unknown	4	22	4	50
17	https://www.youtube.com/watch?v=rs0z7VVf9_c	Robotic Xi Left Upper Lobectomy	May 27, 2016	July 27, 2025	LUL	6281	27	3349	33	1080	1240	Individual surgeon	Unknown	4	22	4	50
18	https://www.youtube.com/watch?v=9e0CoeUhLP4	Robotic right upper lobectomy for lung cancer, Charles Anderson, MD	November 6, 2012	July 27, 2025	RUL	6245	11	4647	5	720	299	Individual surgeon	Unknown	4	22	5	62.5
19	https://www.youtube.com/watch?v=CRIiOCVM53A	Robotic Lobectomy: Review of Anatomy and Technique for RUL, RML, RLL, LUL, and LLL	March 19, 2021	July 27, 2025	RUL, RML, RLL, LUL, and LLL	6009	87	1591	18	1080	78000	Professional society	United States	11	61	3	37.5
20	https://www.youtube.com/watch?v=AaJRQDVnrMU	12-Step Robotic Right Upper Lobectomy	October 17, 2022	July 27, 2025	RUL	5993	47	1013	13	1080	78000	Professional society	United States	15	83	6	75
21	https://www.youtube.com/watch?v=eVHbCeyM-IY	STS University 2019 - Course 11: Robotic Lobectomy	November 7, 2018	July 27, 2025	LUL	5902	38	2454	19	720	13500	Professional society	Unknown	11	61	3	37.5
22	https://www.youtube.com/watch?v=Sg4vQjuQK38	Robotic Lobectomy in 18 Minutes: Step-by-Step, RULobectomy	July 27, 2018	July 27, 2025	RUL	5762	0	2556	18	720	1790	Individual surgeon	Unknown	9	50	5	62.5
23	https://www.youtube.com/watch?v=Bhc-W0_BkrI	Robotic Assisted Left Upper Lobectomy	March 25, 2021	July 27, 2025	LUL	5442	53	1585	39	720	584	Individual surgeon	Unknown	9	50	6	75
24	https://www.youtube.com/watch?v=R2EppC94NRc	Fissureless Robotic Right Lower Lobectomy with step-by-step narration	September 12, 2019	July 27, 2025	RLL	5306	0	2145	35	720	1790	Individual surgeon	Unknown	8	44	5	62.5
25	https://www.youtube.com/watch?v=c45ENlMwXmA	Robotic Lobectomy in 18 Minutes: Step-by-Step, LULobectomy	August 30, 2018	July 27, 2025	LUL	5292	0	2553	18	720	1790	Individual surgeon	Unknown	8	44	5	62.5

Abbreviations: CVS, Critical View of Safety; LAP-VEGaS, Laparoscopic Surgery Video Educational Guidelines; RATS, robotic-assisted thoracic surgery; RML, right middle lobe; STS, science, technology, and society; VATS, video-assisted thoracoscopic surgery; others, see FIGURE 2

**TABLE 2:** Characteristics of the analyzed YouTube videos

Variable	Number (%)
Channel type	
Professional society	8 (32)
Hospital/academic institution	2 (8)
Individual surgeon	13 (52)
Medical industry firm	2 (8)
Country of origin	
United States	8 (32)
India	1 (4)
England	1 (4)
Unknown	15 (60)

**TABLE 3:** Video features and descriptive statistics

Video feature	Median (interquartile range)
Views	7157 (6001-14 151)
Likes	40 (18-91.5)
Days online	2581 (2425-3358)
Video length, min	18 (12-28)

**TABLE 4:** Correlation of video characteristics with the number of views

Dependent variable	Independent variable	*P *value	*R *value	Median (interquartile range)
Views	Lobe	0.43	-	-
	Likes	<0.001	0.639	-
	Days online	0.01	0.5	-
	Video length, min	0.08	-0.355	-
	Image quality, pixel	0.1	-	1080: 10414 (6281-14126) 480: 52501 (29829-73532) 720: 6499 (5762-7794)
	Subscribers	0.33	0.202	-
	Uploader type	0.08	-	Hospital/academic institution: 73532 (52501-94563); Medical industry firm: 18850.5 (14 126-23575); Professional society: 7153.5 (6001-12 282); Individual surgeon: 6499 (5762-9989)
	Country of origin	0.2	-	India: 23575 (1 video); United States: 12069.5 (6579.5-73532); England: 10414 (1 video); Unknown: 6499 (5832-10309.5)

Descriptive statistics were generated for all video characteristics and scoring variables. Normality of distribution was assessed using the Kolmogorov-Smirnov and Shapiro-Wilk tests. Numerical variables were expressed as mean (SD) or median (interquartile range [IQR]), as appropriate. The relationships between numerical variables were assessed using the Spearman correlation analysis. A P value below 0.05 was considered significant.

### Ethics 

As the study was based on publicly accessible surgical videos, ethical approval was not required.

## RESULTS 

A total of 25 videos were included in the final analysis (TABLE 1). The most frequently demonstrated procedure was right upper lobectomy ( n = 12; 48% ), followed by left upper lobectomy ( n = 7; 28% ). Right lower lobectomy was shown in 3 videos ( 12% ), and left lower lobectomy in 2 videos ( 8% ). Only 1 video ( 4% ) included multiple lobectomies (right upper, right middle, right lower, left upper, and left lower; FIGURE 2).

Of the 25 videos evaluated, 13 ( 52% ) were uploaded by individual surgeons, followed by professional societies with 8 videos ( 32% ), while hospital/academic and industry sources each contributed 2 videos (8%). Regarding the country of origin, 8 videos ( 32% ) were from the United States, 1(4%) from India, and 1(4%) from England. Country of origin could not be identified for 60% of the videos due to the absence of explicit information on the channel profiles or descriptions; therefore, these videos were categorized as "unknown." (TABLE 2).

Median (IQR) number of views was 7157 (6001-14151; TABLE 3). A positive correlation was found between the number of views and the number of likes ( R = 0.639; P < 0.001 ). An association was also identified between the number of views and the duration of online availability ( R = 0.5; P = 0.01 ). In contrast, a negative correlation was observed between the number of views and video length, showing a trend toward significance ( R =  − 0.355; P = 0.08 ). There was also a trend toward significance in the association between the number of views and image quality ( P = 0.1 ), and the association between the number of views and uploader type ( P = 0.08 ). No significant association was found between the number of views and the resected lobe, the number of subscribers, or the country of origin (TABLE 4).

Critical View of Safety assessment All 8 CVS criteria were evaluated for each video. Median (IQR) overall CVS compliance rate was 50%(50% − 62.5%). The highest compliance ( 75% ) was observed for the videos ranked as 20 and 23 according to the number of views. As per the CVS criteria, the steps most frequently presented in the videos were bronchial dissection and clear visualization of the pulmonary veins prior to transection. These were followed by lymph node dissection, which was included in many of the videos. Identification of pulmonary artery branches and retrieval of the resected lobe within an endoscopic specimen bag were moderately often demonstrated.

**FIGURE 3:**
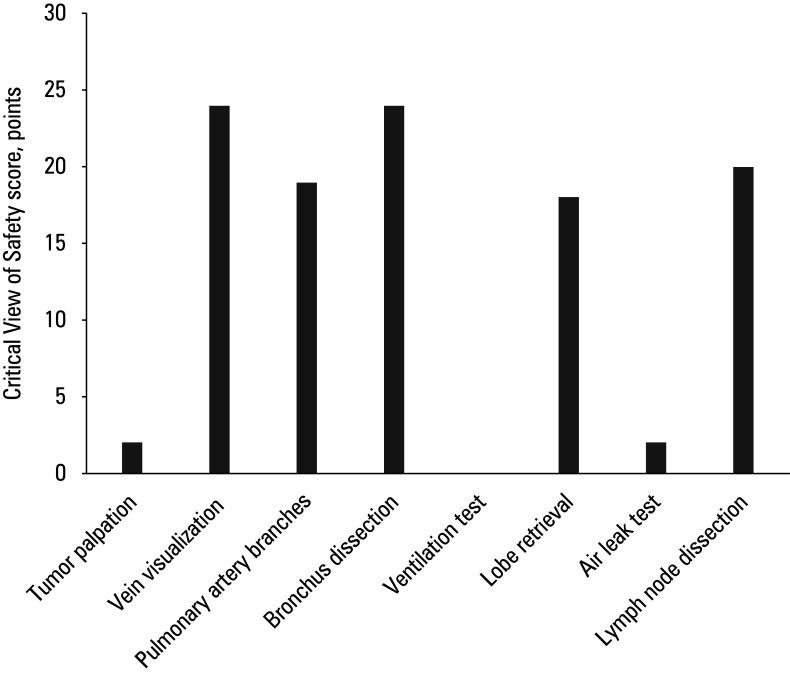
Total score of each Critical View of Safety criterion

In contrast, palpation of the tumor and exploration of the thoracic cavity for additional pathologies were shown only in 2 videos. The most notable deficiencies were observed in the safety verification steps, which are critical for surgical quality. Specifically, the ventilation test prior to bronchial transection was not demonstrated in any of the videos, and the air leak test following bronchial division was presented only in a few (FIGURE 3). These findings indicate that while the videos were successful in demonstrating anatomical structures and the main steps of resection, they frequently neglected complementary measures aimed at verifying surgical safety. No correlation was found between the CVS score and the number of views ( R =  − 0.261; P = 0.21 ), nor between the CVS score and the number of likes ( R =  − 0.25; P = 0.23; TABLE 5).

**TABLE 5:** Correlation between Critical View of Safety/Laparoscopic Surgery Video Educational Guidelines scores and video characteristics

Variable	CVS *R *value	CVS *P *value	LAP-VEGaS *R *value	LAP-VEGaS *P *value
Views	-0.261	0.21	-0.103	0.63
Likes	-0.25	0.23	0.296	0.15

Abbreviations: see TABLE 1

**TABLE 6:** Association between Critical View of Safety scores and video characteristics

Dependent variable	Independent variable	*P* value	*R* value
CVS score	Video length, min	0.58	0.116
	Days online	0.15	-0.297
	Image quality, pixel	0.47	-0.153
	Narration (0-2)	0.18	-
	Uploader type	0.54	-

Abbreviations: see TABLE 1

The correlation analysis showed no association between the CVS score and video length ( R = 0.116; P = 0.58 ), days online ( R =  − 0.297; P = 0.15 ), or image quality ( R =  − 0.153; P = 0.47 ). Similarly, the Kruskal-Wallis tests showed that the narration level ( P = 0.18 ) and uploader type ( P = 0.54 ) were not associated with higher CVS scores TABLE 6.

### Laparoscopic Surgery Video Educational Guidelines conformity 

Compliance with the LAP-VEGaS framework was analyzed using 9 criteria. Median (IQR) LAP-VEGaS score was 8 (4-10.5). According to the item-level analysis based on the LAP-VEGaS checklist, the educational quality of the video content demonstrated substantial heterogeneity. The majority of videos were deemed adequate in terms of presenting the surgical procedure in a standardized step-by-step manner, clearly demonstrating intraoperative findings with consistent anatomical reference. Additionally, image quality and video flow were generally satisfactory, and a considerable portion of the videos included English audio or written narration. The narration was evaluated on a scale of 0 to 2 , where 0 corresponded to no narration, 1 to partial narration, and 2 indicated full narration.

**FIGURE 4:**
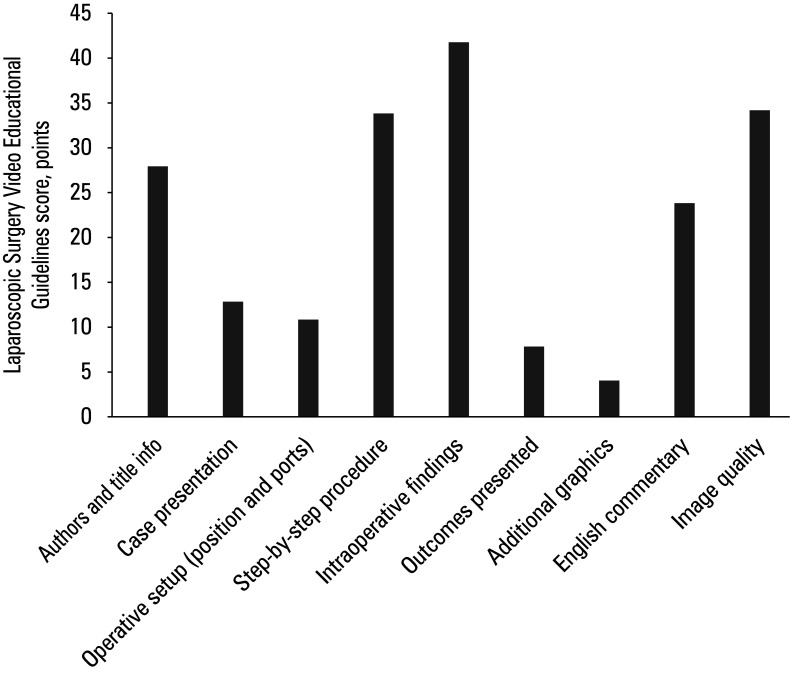
Total score of each Laparoscopic Surgery Video Educational Guidelines criterion

However, several key components that could enhance educational value were frequently omitted. Some videos lacked author or institutional information, and did not include appropriately structured titles reflecting the procedure and underlying pathology. Only a limited number of videos provided a structured case presentation, including patient history, imaging findings, surgical indications, and comorbidities. Similarly, preoperative preparation details-such as patient positioning, port placement, and incision/ extraction site-were absent from most videos. Perioperative outcomes, including operative time, complications, or histopathological results, were rarely reported, and supplementary instructional materials, such as diagrams or anatomical illustrations, were used only in a few videos (FIGURE 4).

Overall, while the videos were effective in demonstrating surgical techniques and anatomy, they largely failed to provide contextual information and perioperative details. This suggests that the videos tend to focus predominantly on technical skill transmission, while complementary content that could enhance educational completeness is often overlooked. There was no correlation between the LAP-VEGaS score and either the number of views ( R =  − 0.103; P = 0.63 ) or the number of likes ( R = 0.296; P = 0.15; TABLE 5).

When the relationship between LAP-VEGaS scores and video characteristics was examined, no correlation was observed with video length ( R =  − 0.231; P = 0.27 ) or image quality ( R = 0.061; P = 0.77 ), whereas a strong negative correlation was identified between days online and LAP-VEGaS scores ( R =  − 0.656; P < 0.001 ). This finding suggests that videos that have been available on the platform for a longer time tend to have lower educational quality. The Kruskal-Wallis test demonstrated a difference in LAP-VEGaS scores across narration levels ( P = 0.001 ), supporting the notion that the presence of narration enhances educational quality. Similarly, 1 -way analysis of variance based on uploader type identified a difference among the groups ( P = 0.002 ). Although homogeneity of variance was confirmed (the Levene test; P = 0.07 ), the Tukey post-hoc analysis did not show pairwise differences, which may be attributed to the small sample size in some uploader categories. Therefore, an additional Mann-Whitney test was performed for only the 2 groups with adequate sample sizes-professional societies vs individual surgeons-which indicated that videos uploaded by professional societies had markedly higher LAP-VEGaS scores ( P = 0.004; TABLE 7).

**TABLE 7:** Association between Laparoscopic Surgery Video Educational Guidelines scores and video characteristics

Dependent variable	Independent variable	*P *value	*R *value
LAP-VEGaS score	Video length, min	0.27	-0.231
	Days online	<0.001	-0.656
	Image quality, pixel	0.77	0.061
	Narration (0-2)	0.001	-
	Uploader type	0.002	-

Abbreviations: see TABLE 1

## DISCUSSION 

This study contributes to the current literature addressing the educational value of RATS lobectomy videos shared on YouTube. Our findings demonstrate that a substantial proportion of these videos fail to meet the minimum standards required for structured surgical training, with notably low compliance with both the CVS and LAP-VEGaS criteria. This observation is consistent with reports from recent investigations in related surgical fields. For example, analyses of laparoscopic cholecystectomy and appendectomy videos have similarly shown that adherence to the LAP-VEGaS and CVS criteria remains as low as 10% − 30%, and that neither view counts nor overall popularity correlate with educational quality.^14^^,^^15^

Similarly, low compliance rates have also been reported in evaluations of other minimally--invasive procedures, such as bariatric surgery and thoracoscopic sympathectomy.^16^^,^^17^

These findings indicate that neither high--resolution imaging nor high view counts guarantee educational quality. Indeed, the literature also underscores that no significant association exists between the number of views, like rates, or video length and the LAP-VEGaS score,^15^^,^^18^ which indicates that relying solely on popularity indicators when selecting videos for surgical learning can be misleading.

RATS lobectomy represents one of the most advanced steps in minimally-invasive thoracic surgery and requires mastery of complex anatomy and 3-dimensional dissection skills. Consequently, the reliability and standardization of video-based education are of critical importance. However, our findings suggest that a considerable proportion of available content lacks essential preoperative information, surgeon identification, patient consent, potential complications, and detailed reporting of key operative steps. Such deficiencies may pose potential risks to both surgical training and patient safety.

In recent years, simulation technologies, virtual/augmented reality, and validated checklists (eg, LAP-VEGaS) have been increasingly recommended in surgical education^19^ These developments have become even more critical as opportunities for live operating room training have diminished. However, our study findings indicate that these standards are not widely implemented and that video platforms lack self-regulatory mechanisms. Therefore, peer-reviewed and curated video libraries developed by professional societies and academic institutions should become essential resources for surgical education.

The strength of our study lies in its focus on an advanced procedure, such as RATS lobectomy, and the evaluation of videos using internationally recognized and validated criteria-CVS and LAP-VEGaS. Despite providing valuable insights, this study has several limitations that should be acknowledged. First, the analysis was restricted to English-language YouTube videos, which may limit the generalizability of the findings. Additionally, as the evaluation reflects the content available at a specific point in time, the results remain susceptible to changes in platform algorithms and video availability. Another limitation is the high proportion of videos from an unknown country of origin, which may reduce the accuracy of geographic comparisons. Furthermore, although the view threshold of 5000 views or more was chosen to ensure relevance by focusing on commonly accessed content, it may have inadvertently excluded newly uploaded but potentially high-quality videos. Finally, data directly related to educational impact and clinical outcomes, such as viewer demographics or learning performance, were beyond the scope of this study and could not be evaluated.

### Teaching points 

A high-quality RATS lobectomy video should clearly demonstrate patient positioning, port placement, and docking. The sequence of vein, artery, and bronchus dissection should be presented in a structured step-by-step manner, and the chosen fissure management strategy (fissure-first or fissure-last) should be explicitly specified and clearly visualized. Systematic hilar and mediastinal lymph node dissection should be thoroughly performed and appropriately illustrated, together with proper techniques for bleeding control and intraoperative troubleshooting. The specimen should be extracted safely, preferably using an endoscopic retrieval bag. Air leak testing, chest tube management, and early postoperative outcomes should also be briefly addressed.

## CONCLUSIONS 

Although YouTube provides an accessible and popular platform for surgical learning, most RATS lobectomy videos do not meet the minimum standards required for structured surgical education. Compliance with the CVS and LAP-VEGaS criteria was found to be low. To ensure safe dissemination of surgical knowledge in thoracic surgery, there is a need for validated and peer-reviewed educational content.

## JOURNAL INFORMATION

Videosurgery and Other Miniinvasive Techniques is an official journal of the Videosurgery Foundation.
